# The Mitochondrial DNA Landscape of Modern Mexico

**DOI:** 10.3390/genes12091453

**Published:** 2021-09-21

**Authors:** Martin Bodner, Ugo A. Perego, J. Edgar Gomez, Ricardo M. Cerda-Flores, Nicola Rambaldi Migliore, Scott R. Woodward, Walther Parson, Alessandro Achilli

**Affiliations:** 1 Institute of Legal Medicine, Medical University of Innsbruck, 6020 Innsbruck, Austria; martin.bodner@i-med.ac.at; 2 Dipartimento di Biologia e Biotecnologie “L. Spallanzani”, Università di Pavia, 27100 Pavia, Italy; uperego@scciowa.edu (U.A.P.); nicola.rambaldi01@universitadipavia.it (N.R.M.); 3 Sorenson Molecular Genealogy Foundation, Salt Lake City, UT 84115, USA; gomeze@familysearch.org (J.E.G.); scott.woodward@gmail.com (S.R.W.); 4 Department of Math and Science, Southeastern Community College, Burlington, IA 52655, USA; 5 FamilySearch Int., Salt Lake City, UT 84150, USA; 6 Facultad de Enfermería, Universidad Autónoma de Nuevo León, Monterrey 64460, Mexico; ricardocerda_mx@yahoo.com.mx; 7 Forensic Science Program, Penn State University, University Park, State College, PA 16802, USA

**Keywords:** forensic science, haplogroups, phylogeny, phylogeography, MtDNA database, quality control, EMPOP

## Abstract

Mexico is a rich source for anthropological and population genetic studies with high diversity in ethnic and linguistic groups. The country witnessed the rise and fall of major civilizations, including the Maya and Aztec, but resulting from European colonization, the population landscape has dramatically changed. Today, the majority of Mexicans do not identify themselves as Indigenous but as admixed, and appear to have very little in common with their pre-Columbian predecessors. However, when the maternally inherited mitochondrial (mt)DNA is investigated in the modern Mexican population, this is not the case. Control region sequences of 2021 samples deriving from all over the country revealed an overwhelming Indigenous American legacy, with almost 90% of mtDNAs belonging to the four major pan-American haplogroups A2, B2, C1, and D1. This finding supports a very low European contribution to the Mexican gene pool by female colonizers and confirms the effectiveness of employing uniparental markers as a tool to reconstruct a country’s history. In addition, the distinct frequency and dispersal patterns of Indigenous American and West Eurasian clades highlight the benefit such large and country-wide databases provide for studying the impact of colonialism from a female perspective and population stratification. The importance of geographical database subsets not only for forensic application is clearly demonstrated.

## 1. Introduction

Mexico, officially the United Mexican States, is the northernmost Central and southernmost North American country. It covers more than 1.9 million km^2^ and is confined by the USA in the North, the Atlantic Ocean in the East, Belize and Guatemala in the South, and the Pacific Ocean in the West. In cultural terms, Mexico is part of Mesoamerica in its center and South and the Greater Southwest and Aridoamerica in its North [1,2,3,4,5,6,7]. It is generally agreed that the first modern human settlers reached America from Asia via Beringia and colonized the double continent in a rapid southward movement after an incubation period [8,9]. Mesoamerica had a pivotal role in this process, providing a bridge and geographic bottleneck into South America and continuous interethnic space. Archaeological evidence has shown human presence in today’s Mexico since 12–15 thousand years (ky) [5,6,10,11,12]. The extensive and arid North and the rainy and narrow central and Southern valleys and mountains required different subsistence strategies: while Northern human populations were hunter-gatherers, Indigenous American (also called Native American) groups in South-Central Mexico began to settle in communities following the development of (maize) agriculture from 7–5 ky ago [2,3,5,13,14]. The following millennia included population movements and tremendous growth in some of these communities, giving rise to complex socio-economic, political, and religious urban centers, which eventually became the foundation of large civilizations, such as the Aztec, Maya, Olmec, Teotihuacan, Toltec, and Zapotec peoples, characterized by relative cultural homogeneity [2,5,15]. Population decline and bottlenecks occurred before, and coincided with, European contact after 1519 CE [15]. While indigenous populations recovered since the 17th century and remained largely isolated until the mid-18th century [14], the demography of Mexico was drastically reshaped following the conquest and colonization. African slaves were brought in as a workforce and the advanced pre-Columbian civilizations were eventually displaced and destroyed [16,17]. Mixed populations increased during the 17th and 18th centuries [18]. The Viceroyalty of New Spain achieved independence from Spain as Mexico in 1821. Today, its population is estimated to be well over 130 million [7], presenting one of the richest ethnic and linguistic diversities of the world [10,19]. The majority of the Mexican population is largely formed by descendants of Indigenous Americans, Spanish (European) immigrants, and African slaves [20]. The meaning of ethnicity has grown out of traditions, and classification can be impacted by different criteria, ranging from cultural, religious, and linguistic heritage, physical appearance to ideology. Self-identified ethnicity may not accurately reflect genetic ancestry [21]. Since census data on ethnicity are not officially collected [7], the proportions and definitions of population groups depend on the method of assessment and vary between sources. In all statistics, around 85–95% of people identified themselves as a mixture of Amerindian and European ancestry, widely called “Mestizos” in the literature, as opposed to the autochthonous population defined by their surviving culture, language, tradition, oral history, and customs [7,10,13,18,20]. A common definition for “Mestizo” is an individual born in Mexico, with a Spanish-derived last name and Mexican ancestors for at least three generations [22]. However, the term is genetically pointless [23] and under criticism. Alternatives include “Amerindian-Spanish”, “(ad)mixed”, “urban”, and “cosmopolitan” Mexicans [7,10,14]. Recent estimates combined the non-Indigenous and non-admixed Mexican population into just 10% “other (mostly European)” [7], and found 2% of the population seeing themselves of African ancestry [24]. Considering language as selection criterion, the Indigenous population proportion is <10% with diminishing tendency. Spanish is the official language and spoken by nearly the entire population, making Mexico the world’s largest Spanish-speaking country by population. Less than 1% of the population speak exclusively indigenous languages while ~6–7% speak some. They are mostly concentrated in Central and Southern Mexico, as before the Spanish conquest [7,13,14,24]. One classification considered 95 indigenous languages in 42 groups and 13 families, plus several “other” languages spoken in Mexico [25]. For these reasons, Mexico has been, and remains a rich source for anthropological and genetic studies, with the maternally inherited mitochondrial (mt)DNA as a classical marker. While some of the very first mitogenetic population studies already included individuals of Mexican descent [26,27,28,29], it has been repeatedly claimed since that country-wide mtDNA data from the general population was underrepresented in databases [13,18,20,30,31,32], together with Indigenous American genetic data in general [33,34]. Previous studies were either based on a small number of subjects (e.g., [35,36]), mtDNA segments shorter than the 1.1 kbp gold-standard control region (CR) range [37] (D-loop, nps 16024–16569 1–576; e.g., [16,18]), ancient DNA [38,39], data from geographically restricted or Indigenous population groups (e.g., [10,40]) where lineage representation could be heavily biased [41], US Mexicans (e.g., [20,21]), did not report the actual haplotypes (e.g., [4,42]), or a combination of these factors. The lack of general Mexico-wide mtDNA data affects both phylo- and population genetic studies aiming to reconstruct the history of human settlement and their interaction [20], as well as the forensic application of mtDNA as a vital niche marker in human identification and the assessment of biogeographic origin [37]. Furthermore, the forensic aspect has increasingly become relevant in lieu of the many unidentified victims attempting to cross the Mexican-US border [43]. To address these limitations, this study presents the first comprehensive overview of the mitogenetic landscape of modern Mexico reporting novel complete mtDNA CR profiles of 2021 individuals collected from the general population across the country.

## 2. Materials and Methods

### 2.1. Sample Collection

**Figure 1:**
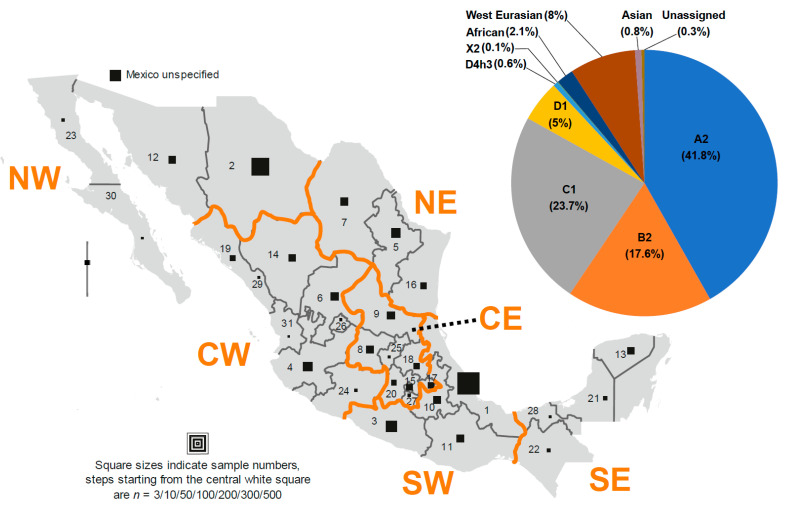
TMA geographic origin and haplogroup classification of the 2021 Mexican individuals. The main figure shows the administrative units of Mexico and the samples from each of the units analyzed in this study. The administrative units are numbered according to Table S1. The geographic subsets indicated by orange lines and text are Northwest (NW), Northeast (NE), Center-West (CW), Center-East (CE), Southwest (SW), and South-East (SE). The pie chart shows the proportions of Indigenous American haplogroups and other contributions to the Mexican mtDNA pool. See text for details.

The study was performed on 2021 mouthwash samples collected from the general Mexican population as part of a worldwide campaign by the Sorenson Molecular Genealogy Foundation (SMGF). All experimental procedures and individual written informed consent, obtained from all donors, were reviewed and approved by the Western Institutional Review Board, Olympia, Washington (USA). Ethno-linguistic affiliation of donors was not recorded. Only individuals where genealogical investigation revealed a terminal maternal ancestor (TMA) from Mexico and unrelatedness were included. The sample set covers all 32 administrative units of Mexico (31 states and Mexico City, the former Federal District), except the easternmost state of Quintana Roo. Sample numbers range from 3 to 511, with a mean and median of 61 and 36, respectively (Table S1). For interpretational purposes, the individuals were combined into macrogeographic subsets, revealing roughly similar sizes: North (seven states, *n* = 567), Center (15 states and Mexico City, *n* = 622), and South (eight states, *n* = 709) (Figure 1). The macro-regions were further divided into Western (Pacific) and Eastern (Atlantic) subsets and sample sizes were again reasonably similar (Northwest, NW: *n* = 388, Northeast, NE: *n* = 179; Centre-West, CW: *n* = 360, Centre-East, CE: *n* = 362), except for the South (Southwest, SW: *n* = 629, Southeast, SE: *n* = 80). The country-wide Western and Eastern datasets contained 1377 and 521 individuals, respectively. A further 123 Mexican individuals were included in the study whose TMA could not be assigned to a specific state for the lack of information or recurrent toponyms (Table S1, Figure 1).

### 2.2. MtDNA Sequencing, Haplogroup Estimation, and Quality Control

Total DNA was extracted using the QIAamp DNA Blood Maxi Kit (Qiagen, Hilden, Germany) according to the manufacturer’s recommendations and stored at −20 °C. The entire control region (CR, nps 16024–16569, 1–576) was amplified and sequenced as described in [44]. Sequence data were assembled and aligned to the revised Cambridge reference sequence (rCRS) [45] using Sequencher v5.4.6 (GeneCodes, Ann Arbor, MI), resulting in haplotypes covering nps 16000–16569 1–580. All mtDNA haplotypes were subjected to forensic quality control, including plausibility checks and phylogenetic inspection on EMPOP (https://empop.online (accessed on 16 August 2021)) [46]. The SAM2 engine [47] implemented in EMPOP was used for phylogenetic haplotype alignment and haplogroup estimation [37] according to the clades cataloged in PhyloTree*mt*, build 17 [48]. SAM2 does not follow a strict classification along a tree but compares the input haplotypes to the large EMPOP dataset of verified complete mitogenomes. According to the variation and fluctuation in this etalon, the haplogroup of the queried haplotype is estimated. Partial mitogenomes, as in this study, might yield several candidate matches within the same cost range of the weighted differences. If so, rather than picking one, the most recent common ancestor (MRCA) haplogroup containing all candidates is provided. This conservative output can form the basis for a further educated haplogroup estimation taking non-genetic information into consideration, such as geographic dispersal and metapopulation [37]. The exact haplogroup can always only be assured by the complete mitogenome. In this study, SAM2 estimates were used (candidates not shown), and the few additional interpretations are explained.

### 2.3. Forensic, Population Genetic, and Phylogeographic Calculations

Forensic and population genetic molecular statistics and diversity indices were calculated from the complete haplotypes using Arlequin v3.5.1.2 [49] disregarding length variation in polycytosine stretches, i.e., using nps 16000–16193 16194–309 310–573 574–580. Random match probability (RMP), power of discrimination (PD, haplotype diversity), and discrimination capacity (DC) were calculated as in [26,50]. A principal component analysis (PCA) to compare the haplogroup frequencies of the Mexican states was performed as previously described [51]. Haplogroup frequency maps were obtained using Surfer v.6.04 (Golden Software, Golden, CO), with the Kriging procedure. Estimates at each grid node were inferred by consideration of the entire data set, as in [52].

## 3. Results

### 3.1. Quality Control

All 2021 haplotypes passed quality control and will be uploaded on the forensic online mtDNA population database EMPOP with reference numbers EMP000849-852 [46]. They constitute the first mtDNA dataset for the general population of contemporary Mexico enabling haplotype and haplogroup frequency and dispersal estimates (Table S2). In 90 individuals (4.4%), one nucleotide, and in four individuals (0.2%), two nucleotides could not unambiguously be determined at altogether 68 different nps. The haplotypes were considered for haplogroup-based analyses since the general high data quality indicated otherwise inconspicuous results. Only the 1927 complete sequences were considered for haplotype-based parameters in order to use their full sequence information.

### 3.2. Forensic and Population Genetic Characterization of the Mexican mtDNA Dataset

The dataset contained 799 different haplotypes in the range nps 16000–16569 1–580, 502 of which (62.8%) were unique, comprising 26.1% of the included individuals. An RMP of 0.5% was calculated, PD reached 99.6%, and DC was 41.5%. The mean number of pairwise differences between two randomly chosen haplotypes (MNPD, k) was 14.995 ± 6.709, resulting in a nucleotide diversity (π) of 0.013 ± 0.006 over the 1168 nps. The most frequent haplotype was 16223T 16290T 16319A 16362C 64T 73G 146C 153G 182T 235G 263G 315.1C 523del 524del, relative to the rCRS [45], belonged to haplogroup A2 and was found 53 times (2.8%). A C1c1 haplotype, 16223T 16298C 16325C 16327T 73G 215G 249del 263G 290del 291del 315.1C 489C, ranked second with 38 observations (2.0%), ahead of a C1c5 haplotype that was found 37 times (1.9%), 16223T 16298C 16325C 16327T 16526A 73G 113T 249del 263G 290del 291del 315.1C 489C (Tables S2–S4). The most and third most frequent haplotypes were just observed once each in the 38,361 worldwide, thereof 17,062 American and 6497 Native American, samples with a range of CR and beyond in EMPOP v4/R13. This yielded haplotype frequency point estimates of 2.6|5.9|15.4 in 100,000 individuals (worldwide|America|Native Americans), respectively. The haplotype ranking second in Mexico was reported 11 times, i.e., at respective frequencies of 2.9|6.4|16.9 in 10,000 (worldwide|America|Native Americans). All 13 hits were made in Native American US populations [46].

### 3.3. The mtDNA Haplogroups of Mexico

The dataset revealed a wide-ranging spectrum of mtDNA haplogroups present in the general country-wide Mexican population (Table 1).

The 2021 individuals were assigned to 170 different named clades (paragroups, haplogroups, and subhaplogroups at different levels) of PhyloTree*mt* build 17 [48] according to their CR sequence. The clades comprised between one (<0.1%) and 501 (24.8%) mtDNAs. The largest clade was A2*, followed by B4b* (*n* = 103; 5.1%) and C1b* (*n* = 99; 4.9%) (Table 1, Figure 1, Table S5). Different macrogeographic TMA origins of Mexicans were revealed when assessing the worldwide dispersal of mtDNA lineages on EMPOP [46]. The predominant proportion of clades was attributable to Indigenous American lineages and altogether comprised 1804 (89.2%) of the individuals. All major pan-American haplogroups (A2, B2, C1b, C1c, C1d, and D1) [53] were highly represented (Figure 1).

The high number of sub-lineages (Table S2) likely reflects the history of settlement, combining multiple populations with different origins, history, and bottlenecks [10]. Haplogroup A2 was the most prevalent with 845 individuals (41.8% of the total) subdivided into 30 different named sub-haplogroups (A2b1, A2d1a, A2e, A2f2, A2f3, A2g, A2h*, A2h1, A2k, A2m, A2p2, A2q*, A2q1, A2r1, A2s, A2u*, A2u1, A2u2, A2v*, A2v1b, A2w1, A2ae, A2af*, A2af1a1, A2af1b2, A2ai, A2aj, A2ak, A2an, A2ao1), unassignable A2* and 10 individuals classified as MRCA-A* (0.5%), where candidate matches indicated A2 status. Haplogroup B2 comprised 356 individuals (17.6%) assigned to B2*, 21 named clades (B2a*, B2a2, B2a3, B2a4a*, B2a4a1, B2b4, B2c2*, B2c2a, B2c2b, B2d, B2g*, B2g1, B2l, B2m, B2o, B2s, B2t, B2v, B2w, B2x, B2y), B4b* with the largest proportion (5.1% of total, i.e., B2* lineages unassignable by CR), and including 0.1% B4* that are likely B4b* haplotypes. Haplogroup C1 comprised 19 clades (C1*, C1b*, C1b1, C1b10, C1b11, C1b14, C1b3, C1b7, C1b8, C1b9, C1c*, C1c1, C1c4, C1c5, C1c6, C1c7, C1d*, C1d1c*, C1d1c1) and 478 (23.7%) individuals. Their largest proportion was assigned to C1b* (4.9%). A further 111 individuals (5.5%) fell into the D1 cluster, including an MRCA-D* singleton, eight MRCA-D4*, and 11 MRCA-M* haplotypes all of which likely belong to D1, 42 D1* mtDNAs (2.1%, the largest proportion within D1) and six named clades (D1f, D1h1, D1h2, D1i*, D1i2, D1m). Additionally, the two rare founder lineages, the pan-American D4h3a and the Northern American X2a [54], were represented in the Mexican dataset by 12 (0.6%; clades D4h3*, D4h3a*, D4h3a3a, D4h3a8) and two (0.1%) individuals, respectively. West Eurasian mtDNA lineages were found in 162 individuals (8.0%) within 48 clades of haplogroups H, HV, J, K, R0, R6, T, and U. The largest haplogroup was H with 16 clades (H*, H1a1c, H1a3b, H1ba, H1bf1, H1c1, H1c3, H1n6, H2a2a1, H2a2b, H5a3, H6, H11a2a, H15*, H15a1b, H82) and 43 individuals (2.1% of all). H comprised 29.6% of the West Eurasian set and, intriguingly, seven individuals (4.3% of the West Eurasian) revealed the most common West Eurasian CR haplotype 16519C 263G 315.1C, which supports a random composition of the sample and demonstrates that the grave forensic implications posed by this haplotype’s frequency of ~3–4% throughout West Eurasia [55] even hold true for Mexico. Sub-Saharan African lineages were found in 42 (2.1%) individuals in 29 clades within haplogroups L0 (L0a1a, L0a2), L1 (L1c1*, L1c1b1, L1c2b2, L1c3a), L2 (L2a1*, L2a1c, L2a5, L2b1a2, L2c*, L2c1, L2c2, L2d, L2e), and L3 (L3*, L3b*, L3b1a9a, L3d*, L3d1b, L3e1*, L3e1a2, L3e2a1, L3e2b, L3e3, L3e5, L3f1b1a, L3f1b4, L3h1a2a). South, Southeast, and East Asian lineages were represented only by four haplogroup F1a1’4 (0.2%) and two haplogroup M (M*, M74a) representatives (0.1%). A further seven (0.3%) mtDNAs estimated to belong to haplogroup R* were not assigned to any macrogeographic region. It is expected that further sequencing would specify their Eurasian origin (Table 1, Figure 1, Tables S2 and S5).

The observed proportions of lineages in this dataset of modern cosmopolitan Mexicans are well in line with reports of self-estimated ancestry as well as mitogenetic data from various admixed population samples that revealed an extremely high Indigenous American prevalence (87–95%, in one non-random outlier 59% [35]) with some West Eurasian (5–6%) and African (1–5%) contribution [13,14,16,18,31]. Mexican US Americans showed slightly lower Indigenous (82–83%) and higher West Eurasian proportions (13–14%), while the African proportion was low (3–4%), which differed from other “Hispanic” groups [20,21]. The breakdown of Indigenous American haplogroups was also in agreement with the previously reported high proportions of haplogroup A (34–51%), intermediate proportions of B (20–28%), C (16–23%), and low proportions of D (6–8%), which were often not further specified, in the general Mexican population [13,16,18,21,30,31]. The numerous investigations on usually small extant Indigenous population samples across Mexico largely delivered the same picture. Almost exclusively, haplogroups A, B, C, and D were found, most often also in this order and at a ratio of coarsely 6:2:(1–2):(0.5–1) [3,4,5,6,10,14,15,17,29,36,40,56,57,58]. On a finer geographic scale, some heterogeneity of frequencies was detected, with other orders and absences [4,6,17,36], likely reflecting lineage drifts in the group’s individual history [10]. Likewise, ancient population samples showed Indigenous lineage patterns similar to the contemporary with some variation [38,59,60,61]. Haplogroups D4h3(a) and X(2a) were occasionally reported [4,10,17,57]. When non-Indigenous lineages were found at all, their combined proportion was usually very low, and nowhere exceeded 14% [3,10,14,29,57,58].

### 3.4. The Phylogeography of mtDNA Haplogroups in Mexico

**Figure 2:**
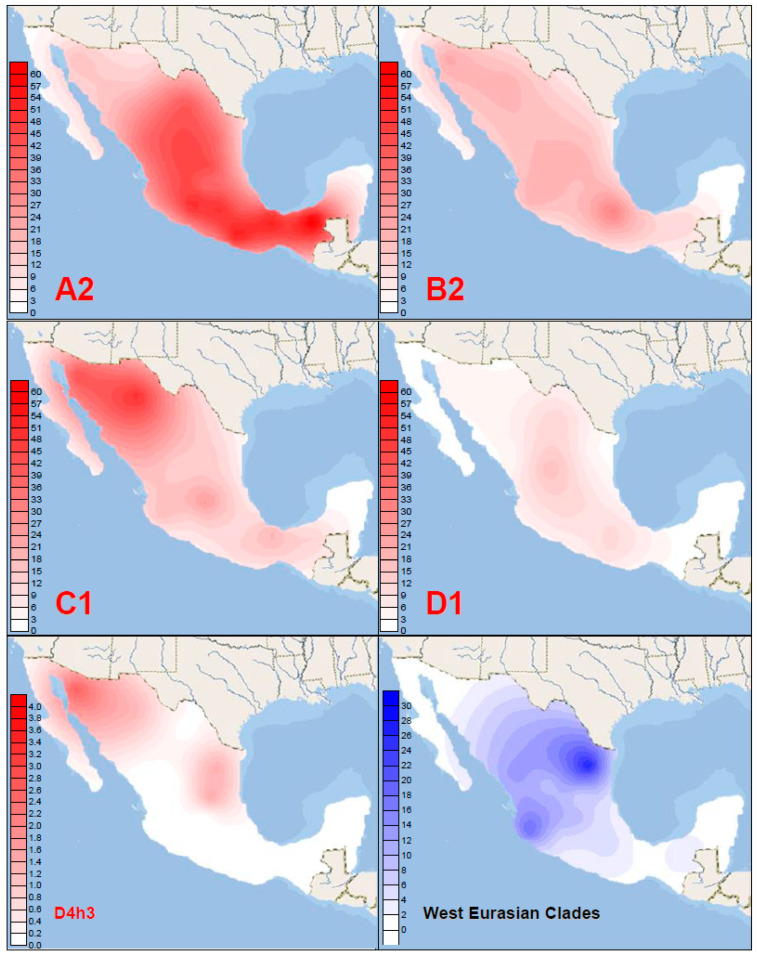
Spatial frequency (%) distributions of Indigenous American haplogroups A2, B2, C1, D1, and D4h3 and the combined West Eurasian lineages in Mexico.

The spatial distribution of both Indigenous American and West Eurasian haplogroup clusters within Mexico was found to be strikingly heterogeneous. The Indigenous lineages showed peculiar and specific patterns along the country. Haplogroup A2 (41.8%) was most frequent in the South, where it comprised 57.0% of individuals, and showed a gradient via the Center (43.6%) to the North (22.2%). Always, the Eastern regions had a higher percentage than the Western parts. Haplogroup C1 (23.7%) presented the opposite pattern: it was particularly frequent in the Northwest (49.5%) and revealed a decline from the North (38.8%) to the Center (16.1%) and South (17.8%) (Table 1, Figure 2).

Both clines were also observed in Indigenous and admixed populations when analyzed at a country-wide scale [2,3,12,14,17,18,62]. These findings illustrate the heterogeneity of lineage dispersal within America, since they contrast with the overall gradients in the double continent [8,18]. Haplogroup B2 (17.6%) was uniformly distributed in the North, Center, and South of Mexico in this sample (16.8–18.5%) with the slightly higher proportion particularly the Northwest (20.4%). A generally higher prevalence in admixed and Indigenous Northern Mexican/Greater Southwest populations has been reported [2,6,14,17,63]. Haplogroup D1 (5.5%) was found to be relatively similarly distributed between the North (3.7%), Center (7.2%), and South (5.4%). Lower frequencies in the North were observed previously [14]. The rare D4h3 (mostly D4h3a) (0.6%) reached 1.6% in the North, particularly Northwest, while elsewhere did not exceed 0.6%. D4h3a spread into the Americas along the Pacific coast and is generally found there [4,10,54]. X2 (likely X2a; 0.1%) is a low-frequency (Northeastern) North American lineage [54] and the X2 haplotype with geographic information in our set was found in the North. The West Eurasian lineages (combined, 8.0%) reached 12.0% in the North, presenting a peak in the Northeast (22.3%) and a southward decline via 10.6% (Center) to 1.6% (South), where the proportion of Indigenous populations is highest. This dispersal was explained by the fact that the main settlement in the North occurred only after European conquest [18,64]. The African lineages (combined, 2.0%) were equally distributed (1.0–3.7%) in all areas except the Southeast, as reported [18] (Table 1, Figure 2).

### 3.5. Regional mtDNA Databases for Mexico?

**Figure 3:**
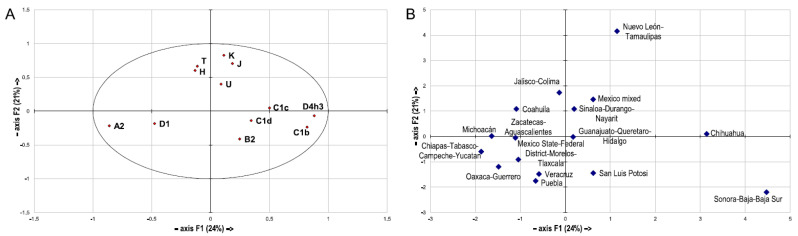
Principal component analysis (PCA) of mtDNA haplogroup profiles of the Mexican administrative units: (**A**) contribution of haplogroups; (**B**) genetic landscape, as depicted by PC1 (axis F1) and PC2 (axis F2). See text for details.

It has been postulated for Indigenous populations of Mexico and beyond that geography rather than linguistic classification predicts mtDNA structure and diversity, suggesting that genetic divergence predates linguistic diversification [2,10,36,63]. It was also described that the general population throughout Mexico mirrors the surrounding Indigenous groups in terms of the mtDNA gene pool composition, particularly in the South [18], supporting geography-based classification. To further evaluate the particular mitophylogeographic structure of Mexico, a PCA comparing the haplogroup frequencies of the different states was performed (Figure 3).

After having reduced the haplogroup complexity to PCs, the first PC was found to primarily mirror the geographic spread, with the Northwestern state group Sonora-Baja California-Baja California del Sur at one, and the Southeast (Chiapas-Tabasco-Campeche-Yucatan) at the other pole. Analysis of the contribution of variables to the first and second PC revealed clear haplogroup structuring. In particular, the second PC separated the Indigenous American from the West Eurasian clades, causing the outgroup position of the Northeastern state group of Nuevo León-Tamaulipas in the PCA (Figure 3). The findings of this study, combined with the literature on the geographically nonuniform dispersal of both Amerindian and West Eurasian haplogroups in Mexico discussed above, thus clearly suggest also evaluating regional subsets within the general population in order to yield accurate dispersal and forensic rarity estimates. Striking regional differences in mtDNA distribution have been described for Argentina, also with a history of European conquest of Indigenous American societies [65]. To investigate this potential for Mexico, database subsets containing the complete Northern (*n* = 545), Central (*n* = 589), and Southern (*n* = 679) haplotypes were analyzed, all surpassing the minimum size considered necessary for a forensic mtDNA database [66] (Tables S4 and S6). The forensic and population genetic parameters calculated from the Northern and Southern subsets were similar to those yielded from the general Mexican database, while the Center was mostly more diverse. The proportion of unique haplotypes among haplotypes|individuals was 60.1–63.9|24.2–27.9% in the general set, North and South, while 71.6|41.1% in the Center; DC was 57.4% in the Center and 40.2–43.7% elsewhere. RMP was calculated as 1.4–1.5% in the North and South, while it was 0.5–0.6% in the Center and general dataset, and PD lay between 98.6% and 99.6%. The MNPD within sets was highest in the North (15.5) and declining via Center (14.8) to South (13.3), and in general 15.00 (Table S4). The corrected MNPD and population pairwise F_ST_ revealed highest differentiation between North and South (1.48|0.094) and lowest between Center and South (0.19|0.014), while the results for the comparison Center-North were intermediate (0.79|0.049), respectively (Tables S4 and S6). Analysis of molecular variance (AMOVA) revealed that the vast majority of the observed variation in the mtDNA structure represented differences within (94.73%) the three subsets and 5.27% were attributable to differences among them (Table S6). The relevance of regional databases was clearly proven by the fundamental differences revealed in the dispersal of haplotypes and singletons, which are of high relevance for forensic statistics [67] (Tables S4 and S7). Around 80% (77.7–83.2) of each subset’s haplotypes were not shared at all among them, and only 15 of the haplotypes (around 0.5% for each subset) were observed in all three subsets. In total, 85.0% (81.6–87.8%) of the unique haplotypes per region were restricted to their region. The most frequent haplotypes over the entire dataset were not homogeneously dispersed along Mexico: the top-ranking haplotype was only found in the South, the second most frequent only in the Center and North, and the third most frequent one only in the North. The most common haplotypes within each regional database were rare or unobserved in the others. Particularly, none of their most frequent haplotypes was observed elsewhere, and the top three Southern haplotypes were only found in that dataset (Tables S4 and S7). This was similarly reported from Uzbekistan, but based on ethnic groups settling in close geographic proximity [68].

### 3.6. The Sex-Biased Genetic History of Mexico

This study demonstrates overwhelming Indigenous American maternal legacy in the extant admixed Mexican population, with almost 90% of mtDNAs belonging to indigenous lineages. A different picture is conveyed by the nuclear genome. Studies on classical blood markers found a ubiquitous European contribution that was, in the North and Center, sometimes larger than the usually predominant Indigenous proportion, while the African proportion was constantly small (references in [13,16,69]). Autosomal microsatellite-based studies revealed an average European ancestry of around 60% in the North, 40% in the Center, and 30% in the South, and 4–8% African contribution [64,70,71]. Investigations of nuclear single nucleotide polymorphisms confirmed the reduced Indigenous ancestry proportion: in admixed populations, the average was 50% in a country-wide sample [72], and more detailed analyses found it lowest in the North (36–51%), 57–66% in the Center, and 59–73% in the South, with a constant African proportion of 2–6% [18,73,74,75]. This striking discrepancy between the two human genomes illustrates the difficulties of categorizing individuals and populations into (self-identified) ancestry categories. Yet, it is coherently explainable by the mode of European conquest that included “directional mating” of immigrant men with indigenous women, causing asymmetric genetic admixture in Indigenous and urban populations in and beyond Mexico [8,13,18,20,29,72,76]. The possibly 250,000 European settlers in Mexico’s colonial period were mostly males, as were the African slaves [16,17]. The European ancestry was determined as mainly Southern European [74]. Consistently, Mexico-wide analyses revealed the paternally inherited Y-chromosomal lineages in admixed populations to be predominantly European (48–89%, more prevalent in the North and West), followed by Indigenous American (13–48%, increased in the Center and Southeast). European paternal lineages were also found in all Indigenous populations studied (1–32%). The African ancestry was relatively the lowest (2–15%) [14,18,69].

## 4. Conclusions

This study provides for the first time a comprehensive overview on the mtDNA variation in the modern general population of Mexico using a large sample covering the entire country. The findings confirm that the genetic impact of European conquest was small in terms of maternal lineage introgression, and the changes in population structure that followed have likely not substantially changed the ample pre-Columbian pattern of mtDNA variation in Mexico. The mitogenetic structure of the general population is still mainly Indigenous. The proportion of West Eurasian mtDNA lineages in the population was found to be low, but with some exceptions, mainly restricted to the Northeast, where the West Eurasian component yielded high proportions. More detailed analyses argued pro regional databases at least for forensic genetic investigations. 

MtDNA is just one genetic marker and, obviously, only a synopsis of all genomic data will fully open the window into the past. This study has also provided insights of a possible sex-differentiated mobility and mixture that impacted cultural as well as biological survival in Mexico, as well as in other countries. The specific insights from the maternal contribution have proven their importance for genetic and anthropological studies aiming to reconstruct and date human settlement. No mitogenome is inherited independently from the nuclear genome. Therefore, this dataset can also help to provide general ancestry estimates for the cosmopolitan population to avoid bias. Understanding population stratification, genetic contributions, and their association with medical traits in admixed populations and individuals is highly relevant to biomedical research and personalized medicine [13,74,77,78].

## Figures and Tables

**Table 1:** MtDNA haplogroups in 2021 Mexican individuals. Proportions [%] of Indigenous American haplogroups and other contributions to the Mexican mtDNA pool in the 2021 individuals included in this study ^1^ and geographic subsets ². See text for details.

	Mexico	North	NW	NE	Center	CW	CE	South	SW	SE	West	East
n	2021	567	388	179	622	360	262	709	629	80	1377	521
A2	41.8	22.2	14.7	38.5	43.6	42.5	45.0	57.0	55.5	68.8	40.6	46.4
B2	17.6	18.5	20.4	14.5	18.3	17.5	19.5	16.8	17.3	12.5	18.2	16.7
C1	23.7	38.8	49.5	15.6	16.1	14.2	18.7	17.8	18.4	12.5	26.1	16.7
D1	5.5	3.7	3.1	5.0	7.2	6.9	7.6	5.4	5.9	1.3	5.4	5.8
D4h3(a)	0.6	1.6	1.8	1.1	0.2	--	0.4	0.1	0.2	--	0.6	0.6
X2(a)	0.1	0.0	0.0	--	--	--	--	--	--	--	0.0	--
West Eurasian	8.0	12.0	7.2	22.3	10.6	13.6	6.5	1.6	1.6	1.3	6.3	11.1
African	2.1	2.1	1.8	2.8	3.7	5.0	1.9	1.0	1.1	--	2.3	1.9

^1^ “--” indicates absence, while 0.0 is a low percentage rounded. ² NW—Northwest, NE—Northeast, CW—Center-West, CE—Center-East, SW—Southwest, SE—Southeast.

## Data Availability

The haplotypes will be uploaded to the online forensic mtDNA population database EMPOP (https://empop.online (accessed on 16 August 2021)) (EMP000849-852).

## Supplementary Materials

The following are available online at https://www.mdpi.com/article/10.3390/genes12091453/s1. Table S1: TMA geographic origin of the 2021 Mexican individuals included in this study by administrative unit, Table S2: The 2021 Mexican individuals included in this study including TMA origin and mtDNA haplogroup, Table S3: Haplotype frequencies in the Mexican dataset, Table S4: Intra-population forensic and population genetic parameters calculated from the Mexican dataset and subsets, Table S5: Haplogroup frequencies in the Mexican dataset, Table S6: Inter-population forensic and population genetic parameters calculated from the Mexican dataset subsets, Table S7: Haplotype frequencies in the Mexican database subsets and shared haplotypes among them.

## References

[1] UN Department of Economic and Social Affairs Statistics Division (1999). Standard Country or Area Codes for Statistical Use.

[2] Kemp B.M., Gonzalez-Oliver A., Malhi R.S., Monroe C., Schroeder K.B., McDonough J., Rhett G., Resendez A., Penaloza-Espinosa R.I., Buentello-Malo L. (2010). Evaluating the farming/language dispersal hypothesis with genetic variation exhibited by populations in the Southwest and Mesoamerica. Proc. Natl. Acad. Sci. USA.

[3] Gorostiza A., Acunha-Alonzo V., Regalado-Liu L., Tirado S., Granados J., Samano D., Rangel-Villalobos H., Gonzalez-Martin A. (2012). Reconstructing the history of Mesoamerican populations through the study of the mitochondrial DNA control region. PLoS ONE.

[4] Mizuno F., Gojobori J., Wang L., Onishi K., Sugiyama S., Granados J., Gomez-Trejo C., Acuna-Alonzo V., Ueda S. (2014). Complete mitogenome analysis of indigenous populations in Mexico: Its relevance for the origin of Mesoamericans. J. Hum. Genet..

[5] Gonzalez-Martin A., Gorostiza A., Regalado-Liu L., Arroyo-Pena S., Tirado S., Nuno-Arana I., Rubi-Castellanos R., Sandoval K., Coble M.D., Rangel-Villalobos H. (2015). Demographic history of indigenous populations in Mesoamerica based on mtDNA sequence data. PLoS ONE.

[6] Morales-Arce A.Y., Hofman C.A., Duggan A.T., Benfer A.K., Katzenberg M.A., McCafferty G., Warinner C. (2017). Successful reconstruction of whole mitochondrial genomes from ancient Central America and Mexico. Sci. Rep..

[7] (2021). Central Intelligence Agency: The World Factbook 2021.

[8] Schurr T.G. (2004). The peopling of the New World: Perspectives from molecular anthropology. Annu. Rev. Anthropol..

[9] Bodner M., Perego U.A., Huber G., Fendt L., Rock A.W., Zimmermann B., Olivieri A., Gomez-Carballa A., Lancioni H., Angerhofer N. (2012). Rapid coastal spread of first americans: Novel insights from South America’s Southern Cone mitochondrial genomes. Genome Res..

[10] Sandoval K., Buentello-Malo L., Penaloza-Espinosa R., Avelino H., Salas A., Calafell F., Comas D. (2009). Linguistic and maternal genetic diversity are not correlated in native Mexicans. Hum. Genet..

[11] Chatters J.C., Kennett D.J., Asmerom Y., Kemp B.M., Polyak V., Blank A.N., Beddows P.A., Reinhardt E., Arroyo-Cabrales J., Bolnick D.A. (2014). Late pleistocene human skeleton and mtDNA link Paleoamericans and modern Native Americans. Science.

[12] Capodiferro M.R., Aram B., Raveane A., Migliore N.R., Colombo G., Ongaro L., Rivera J., Mendizabal T., Hernandez-Mora I., Tribaldos M. (2021). Archaeogenomic distinctiveness of the Isthmo-Colombian area. Cell.

[13] Guardado-Estrada M., Juarez-Torres E., Medina-Martinez I., Wegier A., Macias A., Gomez G., Cruz-Talonia F., Roman-Bassaure E., Pinero D., Kofman-Alfaro S. (2009). A great diversity of Amerindian mitochondrial DNA ancestry is present in the Mexican Mestizo population. J. Hum. Genet..

[14] Gonzalez-Sobrino B.Z., Pintado-Cortina A.P., Sebastian-Medina L., Morales-Mandujano F., Contreras A.V., Aguilar Y.E., Chavez-Benavides J., Carrillo-Rodriguez A., Silva-Zolezzi I., Medrano-Gonzalez L. (2016). Genetic diversity and differentiation in urban and indigenous populations of Mexico: Patterns of mitochondrial DNA and Y-chromosome lineages. Biodemogr. Soc. Biol..

[15] Gojobori J., Mizuno F., Wang L., Onishi K., Granados J., Gomez-Trejo C., Acuna-Alonzo V., Ueda S. (2015). MtDNA diversity of the Zapotec in Mexico suggests a population decline long before the first contact with Europeans. J. Hum. Genet..

[16] Green L.D., Derr J.N., Knight A. (2000). MtDNA affinities of the peoples of North-Central Mexico. Am. J. Hum. Genet..

[17] Gonzalez-Oliver A., Pineda-Vazquez D., Garfias-Morales E., La Cruz-Laina I., Medrano-Gonzalez L., Marquez-Morfin L., Ortega-Munoz A. (2018). Genetic overview of the Maya populations: Mitochondrial DNA haplogroups. Hum. Biol..

[18] Martinez-Cortes G., Salazar-Flores J., Haro-Guerrero J., Rubi-Castellanos R., Velarde-Felix J.S., Munoz-Valle J.F., Lopez-Casamichana M., Carrillo-Tapia E., Canseco-Avila L.M., Bravi C.M. (2013). Maternal admixture and population structure in Mexican-Mestizos based on mtDNA haplogroups. Am. J. Phys. Anthropol..

[19] Vidal O., Brusca R.C. (2020). Mexico’s biocultural diversity in peril. Rev. Biol. Trop..

[20] Kumar S., Bellis C., Zlojutro M., Melton P.E., Blangero J., Curran J.E. (2011). Large scale mitochondrial sequencing in Mexican Americans suggests a reappraisal of Native American origins. BMC Evol. Biol.

[21] Mitchell S.L., Goodloe R., Brown-Gentry K., Pendergrass S.A., Murdock D.G., Crawford D.C. (2014). Characterization of mitochondrial haplogroups in a large population-based sample from the United States. Hum. Genet..

[22] Gorodezky C., Alaez C., Vazquez-Garcia M.N., de la Rosa G., Infante E., Balladares S., Toribio R., Perez-Luque E., Munoz L. (2001). The genetic structure of Mexican Mestizos of different locations: Tracking back their origins through MHC genes, blood group systems, and microsatellites. Hum. Immunol..

[23] Salas A., Acosta A., Alvarez-Iglesias V., Cerezo M., Phillips C., Lareu M.V., Carracedo A. (2008). The mtdna ancestry of admixed Colombian populations. Am. J. Hum. Biol..

[24] Instituto Nacional de Estadística y Geografía (México) (2021). Panorama Sociodemográfico de México—Censo de Población y Vivienda 2020.

[25] Instituto Nacional de Estadística y Geografía (México) (2016). Clasificación de Lenguas Indígenas 2010.

[26] Stoneking M., Hedgecock D., Higuchi R.G., Vigilant L., Erlich H.A. (1991). Population variation of human mtDNA control region sequences detected by enzymatic amplification and sequence-specific oligonucleotide probes. Am. J. Hum. Genet..

[27] Horai S., Kondo R., Nakagawahattori Y., Hayashi S., Sonoda S., Tajima K. (1993). Peopling of the america, founded by 4 major lineages of mitochondrial DNA. Mol. Biol. Evol..

[28] Torroni A., Schurr T.G., Cabell M.F., Brown M.D., Neel J.V., Larsen M., Smith D.G., Vullo C.M., Wallace D.C. (1993). Asian affinities and continental radiation of the 4 founding native-american mtDNAs. Am. J. Hum. Genet..

[29] Torroni A., Chen Y.S., Semino O., Santachiarabeneceretti A.S., Scott C.R., Lott M.T., Winter M., Wallace D.C. (1994). MtDNA and Y-chromosome polymorphisms in 4 native-american populations from Southern Mexico. Am. J. Hum. Genet..

[30] Bonilla C., Gutierrez G., Parra E.J., Kline C., Shriver M.D. (2005). Admixture analysis of a rural population of the state of Guerrero, Mexico. Am. J. Phys. Anthropol..

[31] Rosas R.C.B., Sesma A.M., Ortega L.H., Gonzalez L.H., Avalos J.V., Cruz A.A.A. (2019). The utility of genomic public databases to mitochondrial haplotyping in contemporary Mestizo population of Mexican origin. Mitochondrial DNA A.

[32] Parra J.C.V., Rosas R.C.B., Leon D.C.Y. (2018). Inequality in medical-genetic research towards minority groups an approach to the case of people of African descent in Mexico. Res. J. Med. Sci..

[33] Avila-Arcos M.C., McManus K.F., Sandoval K., Rodriguez-Rodriguez J.E., Villa-Islas V., Martin A.R., Luisi P., Penaloza-Espinosa R.I., Eng C., Huntsman S. (2020). Population history and gene divergence in Native Mexicans inferred from 76 human exomes. Mol. Biol. Evol..

[34] Aguilar-Ordonez I., Perez-Villatoro F., Garcia-Ortiz H., Barajas-Olmos F., Ballesteros-Villascan J., Gonzalez-Buenfil R., Fresno C., Garciarrubio A., Fernandez-Lopez J.C., Tovar H. (2021). Whole genome variation in 27 Mexican indigenous populations, demographic and biomedical insights. PLoS ONE.

[35] Campos-Sanchez R., Barrantes R., Silva S., Escamilla M., Ontiveros A., Nicolini H., Mendoza R., Munoz R., Raventos H. (2006). Genetic structure analysis of three Hispanic populations from Costa Rica, Mexico, and the Southwestern United States using Y-chromosome STR markers and mtDNA sequences. Hum. Biol..

[36] Gonzalez-Oliver A., Garfias-Morales E., Smith D.G., Quinto-Sanchez M. (2017). Mitochondrial DNA analysis of Mazahua and Otomi indigenous populations from Estado de Mexico suggests a distant common ancestry. Hum. Biol..

[37] Parson W., Gusmao L., Hares D.R., Irwin J.A., Mayr W.R., Morling N., Pokorak E., Prinz M., Salas A., Schneider P.M. (2014). DNA commission of the International Society for Forensic Genetics: Revised and extended guidelines for mitochondrial DNA typing. Forensic Sci. Int. Genet..

[38] Kemp B.M., Reséndez A., Berrelleza J.A.R.B., Malhi R.S., Smith D.G., Reed D.M. (2005). An analysis of ancient aztec mtdna from tlatelolco: Pre-Columbian relations and the spread of Uto-Aztecan. Biomolecular Archaeology: Genetic Approaches to the Past.

[39] Alvarez-Sandoval B.A., Manzanilla L.R., Gonzalez-Ruiz M., Malgosa A., Montiel R. (2015). Genetic evidence supports the multiethnic character of Teopancazco, a neighborhood center of Teotihuacan, Mexico (AD 200-600). PLoS ONE.

[40] Watkins W.S., Xing J.C., Huff C., Witherspoon D.J., Zhang Y.H., Perego U.A., Woodward S.R., Jorde L.B. (2012). Genetic analysis of ancestry, admixture and selection in Bolivian and Totonac populations of the New World. BMC Genet..

[41] Cardoso S., Alfonso-Sanchez M.A., Valverde L., Sanchez D., Zarrabeitia M.T., Odriozola A., Martinez-Jarreta B., de Pancorbo M.M. (2012). Genetic uniqueness of the Waorani tribe from the Ecuadorian Amazon. Heredity.

[42] Mizuno F., Kumagai M., Kurosaki K., Hayashi M., Sugiyama S., Ueda S., Wang L. (2017). Imputation approach for deducing a complete mitogenome sequence from low-depth-coverage next-generation sequencing data: Application to ancient remains from the Moon pyramid, Mexico. J. Hum. Genet..

[43] Migration Data Portal: Migrant Deaths and Disappearances. https://migrationdataportal.org/themes/migrant-deaths-and-disappearances.

[44] Perego U.A., Lancioni H., Tribaldos M., Angerhofer N., Ekins J.E., Olivieri A., Woodward S.R., Pascale J.M., Cooke R., Motta J. (2012). Decrypting the mitochondrial gene pool of modern Panamanians. PLoS ONE.

[45] Andrews R.M., Kubacka I., Chinnery P.F., Lightowlers R.N., Turnbull D.M., Howell N. (1999). Reanalysis and revision of the Cambridge Reference Sequence for human mitochondrial DNA. Nat. Genet..

[46] Parson W., Dür A. (2007). EMPOP-a forensic mtDNA database. Forensic Sci. Int. Genet..

[47] Huber N., Parson W., Dür A. (2018). Next generation database search algorithm for forensic mitogenome analyses. Forensic Sci. Int. Genet..

[48] Van Oven M., Kayser M. (2009). Updated comprehensive phylogenetic tree of global human mitochondrial DNA variation. Hum. Mutat..

[49] Excoffier L., Lischer H.E. (2010). Arlequin suite ver 3.5: A new series of programs to perform population genetics analyses under Linux and Windows. Mol. Ecol. Resour..

[50] Kiesler K.M., Coble M.D., Hall T.A., Vallone P.M. (2014). Comparison of base composition analysis and sanger sequencing of mitochondrial DNA for four U.S. Population groups. Forensic Sci. Int. Genet..

[51] Modi A., Lancioni H., Cardinali I., Capodiferro M.R., Rambaldi Migliore N., Hussein A., Strobl C., Bodner M., Schnaller L., Xavier C. (2020). The mitogenome portrait of Umbria in Central Italy as depicted by contemporary inhabitants and pre-Roman remains. Sci. Rep..

[52] Olivieri A., Sidore C., Achilli A., Angius A., Posth C., Furtwangler A., Brandini S., Capodiferro M.R., Gandini F., Zoledziewska M. (2017). Mitogenome diversity in Sardinians: A genetic window onto an island’s past. Mol. Biol. Evol..

[53] Perego U.A., Angerhofer N., Pala M., Olivieri A., Lancioni H., Hooshiar Kashani B., Carossa V., Ekins J.E., Gomez-Carballa A., Huber G. (2010). The initial peopling of the Americas: A growing number of founding mitochondrial genomes from Beringia. Genome Res..

[54] Perego U.A., Achilli A., Angerhofer N., Accetturo M., Pala M., Olivieri A., Hooshiar Kashani B., Ritchie K.H., Scozzari R., Kong Q.P. (2009). Distinctive paleo-indian migration routes from Beringia marked by two rare mtDNA haplogroups. Curr. Biol..

[55] Bodner M., Iuvaro A., Strobl C., Nagl S., Huber G., Pelotti S., Pettener D., Luiselli D., Parson W. (2015). Helena, the hidden beauty: Resolving the most common West Eurasian mtdna control region haplotype by massively parallel sequencing an Italian population sample. Forensic Sci. Int. Genet..

[56] Lorenz J.G., Smith D.G. (1996). Distribution of four founding mtDNA haplogroups among Native North Americans. Am. J. Phys. Anthropol..

[57] Penaloza-Espinosa R.I., Arenas-Aranda D., Cerda-Flores R.M., Buentello-Malo L., Gonzalez-Valencia G., Torres J., Alvarez B., Mendoza I., Flores M., Sandoval L. (2007). Characterization of mtDNA haplogroups in 14 Mexican indigenous populations. Hum. Biol..

[58] Sanchez-Boiso A., Penaloza-Espinosa R.I., Castro-Sierra E., Cerda-Flores R.M., Buentello-Malo L., Sanchez-Urbina R., Ortiz-de-Luna R.I., Rodriguez-Espino B.A., Salamanca-Gomez F.A., Flores-Ayon M.P. (2011). Genetic structure of three native Mexican communities based on mtDNA haplogroups, and ABO and Rh blood group systems. Rev. Investig. Clin..

[59] Gonzalez-Oliver A., Marquez-Morfin L., Jimenez J.C., Torre-Blanco A. (2001). Founding amerindian mitochondrial DNA lineages in ancient Maya from Xcaret, Quintana Roo. Am. J. Phys. Anthropol..

[60] Mata-Miguez J., Overholtzer L., Rodriguez-Alegria E., Kemp B.M., Bolnick D.A. (2012). The genetic impact of Aztec imperialism: Ancient mitochondrial DNA evidence from Xaltocan, Mexico. Am. J. Phys. Anthropol..

[61] Ochoa-Lugo M.I., Munoz M.L., Perez-Ramirez G., Beaty K.G., Lopez-Armenta M., Cervini-Silva J., Moreno-Galeana M., Meza A.M., Ramos E., Crawford M.H. (2016). Genetic affiliation of pre-Hispanic and contemporary Mayas through maternal linage. Hum. Biol..

[62] Monroe C., Kemp B.M., Smith D.G. (2013). Exploring prehistory in the North American Southwest with mitochondrial DNA diversity exhibited by Yumans and Athapaskans. Am. J. Phys. Anthropol..

[63] Malhi R.S., Mortensen H.M., Eshleman J.A., Kemp B.M., Lorenz J.G., Kaestle F.A., Johnson J.R., Gorodezky C., Smith D.G. (2003). Native american mtDNA prehistory in the American Southwest. Am. J. Phys. Anthropol..

[64] Rubi-Castellanos R., Martinez-Cortes G., Munoz-Valle J.F., Gonzalez-Martin A., Cerda-Flores R.M., Anaya-Palafox M., Rangel-Villalobos H. (2009). Pre-Hispanic Mesoamerican demography approximates the present-day ancestry of Mestizos throughout the territory of Mexico. Am. J. Phys. Anthropol..

[65] Bobillo M.C., Zimmermann B., Sala A., Huber G., Rock A., Bandelt H.J., Corach D., Parson W. (2010). Amerindian mitochondrial DNA haplogroups predominate in the population of Argentina: Towards a first nationwide forensic mitochondrial DNA sequence database. Int. J. Leg. Med..

[66] Gusmao L., Butler J.M., Linacre A., Parson W., Roewer L., Schneider P.M., Carracedo A. (2017). Revised guidelines for the publication of genetic population data. Forensic Sci. Int. Genet..

[67] Brenner C.H. (2010). Fundamental problem of forensic mathematics-the evidential value of a rare haplotype. Forensic Sci. Int. Genet..

[68] Irwin J.A., Ikramov A., Saunier J., Bodner M., Amory S., Rock A., O’Callaghan J., Nuritdinov A., Atakhodjaev S., Mukhamedov R. (2010). The mtDNA composition of Uzbekistan: A microcosm of Central Asian patterns. Int. J. Leg. Med..

[69] Rangel-Villalobos H., Munoz-Valle J.F., Gonzalez-Martin A., Gorostiza A., Magana M.T., Paez-Riberos L.A. (2008). Genetic admixture, relatedness, and structure patterns among Mexican populations revealed by the Y-chromosome. Am. J. Phys. Anthropol..

[70] Salazar-Flores J., Zuniga-Chiquette F., Rubi-Castellanos R., Alvarez-Miranda J.L., Zetina-Hernandez A., Martinez-Sevilla V.M., Gonzalez-Andrade F., Corach D., Vullo C., Alvarez J.C. (2015). Admixture and genetic relationships of Mexican Mestizos regarding Latin American and Caribbean populations based on 13 CODIS-STRs. Homo.

[71] Cerda-Flores R.M., Budowle B., Jin L., Barton S.A., Deka R., Chakraborty R. (2002). Maximum likelihood estimates of admixture in Northeastern Mexico using 13 short tandem repeat loci. Am. J. Hum. Biol..

[72] Bryc K., Velez C., Karafet T., Moreno-Estrada A., Reynolds A., Auton A., Hammer M., Bustamante C.D., Ostrer H. (2010). Genome-wide patterns of population structure and admixture among Hispanic/Latino populations. Proc. Natl. Acad. Sci. USA.

[73] Silva-Zolezzi I., Hidalgo-Miranda A., Estrada-Gil J., Fernandez-Lopez J.C., Uribe-Figueroa L., Contreras A., Balam-Ortiz E., del Bosque-Plata L., Velazquez-Fernandez D., Lara C. (2009). Analysis of genomic diversity in Mexican Mestizo populations to develop genomic medicine in Mexico. Proc. Natl. Acad. Sci. USA.

[74] Moreno-Estrada A., Gignoux C.R., Fernandez-Lopez J.C., Zakharia F., Sikora M., Contreras A.V., Acuna-Alonzo V., Sandoval K., Eng C., Romero-Hidalgo S. (2014). Human genetics. The genetics of Mexico recapitulates Native American substructure and affects biomedical traits. Science.

[75] Huerta-Chagoya A., Moreno-Macias H., Fernandez-Lopez J.C., Ordonez-Sanchez M.L., Rodriguez-Guillen R., Contreras A., Hidalgo-Miranda A., Alfaro-Ruiz L.A., Salazar-Fernandez E.P., Moreno-Estrada A. (2019). A panel of 32 AIMs suitable for population stratification correction and global ancestry estimation in Mexican Mestizos. BMC Genet..

[76] Lopopolo M., Borsting C., Pereira V., Morling N. (2016). A study of the peopling of Greenland using next generation sequencing of complete mitochondrial genomes. Am. J. Phys. Anthropol..

[77] Belbin G.M., Nieves-Colon M.A., Kenny E.E., Moreno-Estrada A., Gignoux C.R. (2018). Genetic diversity in populations across Latin America: Implications for population and medical genetic studies. Curr. Opin. Genet. Dev..

[78] Colistro V., Rojas-Martinez A., Carracedo A., Tomlinson I., Carvajal-Carmona L., Cruz R., Sans M., CHIBCA Consortium (2021). CHIBCA Consortium; Population structure and relatedness estimates in a Mexican sample. Ann. Hum. Genet..

[79] D’Amato M.E., Bodner M., Butler J.M., Gusmao L., Linacre A., Parson W., Schneider P.M., Vallone P., Carracedo A. (2020). Ethical publication of research on genetics and genomics of biological material: Guidelines and recommendations. Forensic Sci. Int. Genet..

